# Baseline Serum Estradiol Level Is Associated with Acute Kidney Injury in Patients with Moderately Severe and Severe Acute Pancreatitis

**DOI:** 10.1155/2022/2623199

**Published:** 2022-06-25

**Authors:** Jia-Jia Pan, Wei-Li Liu, Guo-Tao Lu, Xing-Jie Ma, Qing-Bin Zheng, Guang-Fa Wei, Ge-Yan Tian, Li-Jun Meng

**Affiliations:** ^1^Department of Intensive Care, The Affiliated Hospital of Yangzhou University, 368 Hanjiang Road, Yangzhou City, Jiangsu Province 225100, China; ^2^Department of Gastroenterology, The Affiliated Hospital of Yangzhou University, 368 Hanjiang Road, Yangzhou City, Jiangsu Province 225100, China

## Abstract

**Background:**

Sexual dimorphism with critical diseases has been documented. However, the role of serum sex hormones for the presence of acute kidney injury (AKI) in moderately or severe acute pancreatitis (MSAP and SAP) patients remains controversial. Here we set out to evaluate whether early (first 48 h) serum estradiol level is associated with AKI in patients with MSAP and SAP. *Patients and Methods*. We retrospectively collected data from patients with preliminary diagnosis of MSAP and SAP from the Affiliated Hospital of Yangzhou University between January 2014 and June 2018. Serum sex hormones were extracted for further assessment within first 48 h following admission. Logistic regression analysis and the receiving operating characteristic (ROC) curve were applied to evaluate the association and correlation between serum sex hormones and AKI.

**Results:**

Data from a total of 122 patients with MSAP or SAP were enrolled in this study. There were no differences in the incidence of AKI between males and females. However, comparing with patients without AKI, those with AKI saw higher estradiol level (*p* ≤ 0.01) and slight higher progesterone level (*p* = 0.014) but similar testosterone level (*p* = 0.668). Interestingly, during both the manual selection and the stepwise backward logistic regression analysis, serum estradiol level was independently associated with AKI in patients with MSAP and SAP (OR 4.699, CI 1.783-12.386, and *p* = 0.002). Additionally, area under the curve of ROC (AUCROC) showed that serum estradiol level was a proper predictor for AKI (area under the curve 0.875). Specifically, the serum estradiol level of 223.15 pg/mL demonstrated a 92.3% sensitive and a 79.3% specificity in predicting AKI of MSAP and SAP patients, respectively.

**Conclusions:**

High baseline serum estradiol level appears to be an independent risk factor for AKI in patients with MSAP and SAP. It also tends to be an appropriate indicator for AKI.

## 1. Introduction

Both moderately severe and severe acute pancreatitis (MSAP and SAP) are clinically serious diseases. Despite recent therapeutic progresses, they seem continuously to be associated with high morbidity and mortality rates [1]. Acute kidney injury (AKI), one of the major complications of MSAP and SAP, is associated with elevated in-hospital mortality and morbidity [2]. Recent studies have reported a fivefold increase in mortality of SAP patients with AKI (66.6%) after comparing with SAP patients without AKI (14.5%) [3]. Therefore, early identification of whether patients are likely or unlikely to develop AKI may assist physicians towards their decision on appropriate treatment.

Sexual dimorphism in trauma-hemorrhagic shock and sepsis has been well documented in both animal and clinical studies [4]. Besides, it has also been established but not well understood in the development of AKI. Animal models have indicated a protective effect of female sex on the development of AKI [5, 6]. Contrary to these experimental observations, the direction of sexual dimorphism has been reported to be controversial during AKI in human. Chai et al. [7] demonstrated that male patients with acute pancreatitis were more likely to develop AKI. Interestingly, Feng et al. demonstrated that serum estradiol was an independent predictor of AKI in patients with septic shock [8]. Therefore, the association between serum estradiol and AKI in patients with MSAP and SAP remains unclear and inclusive. In this study, we hypothesized that a high baseline serum estradiol level would be associated with high incidence of AKI in patients with MSAP and SAP.

## 2. Patients and Methods

### 2.1. Participants

We retrospectively collected data from the Affiliated Hospital of Yangzhou University from January 2014 to June 2018. A total of 122 patients with MSAP and SAP who admitted to and stayed in the hospital for at least 48 hours were enrolled in this study. AP diagnosis was based on the criteria proposed by the Revised Atlanta Classification [9], in which two out of the following three criteria need to be fulfilled: (1) acute characteristic of upper abdominal pain; (2) serum amylase is three times higher than the upper limit; and (3) observations of AP on CT scan, abdominal ultrasound, or MRI. Moderate severe pancreatitis was defined as transient organ failure (<48 h) with or without local complications, and severe pancreatitis was defined as persistent organ failure (≥48 h). Pregnant women, patients with preexisting chronic renal failure and those younger than 18 were excluded here. All patients received standardized treatment according to treatment guidelines for acute pancreatitis [10].

### 2.2. Data Collection

Demographic characteristics including age, gender, in-hospital mortality, and etiology of MSAP and SAP were recorded. The APACHE II score, multiple organ dysfunction score (MODS) [11], and sequential organ failure assessment (SOFA) score [12] were also collected at the time of admission to evaluate the severity of the disease. AKI was diagnosed and classified using the Kidney Disease Improving Global Outcomes (KDIGO) consensus criteria [13], which stratifies AKI into 3 stages: stage 1 (1.5 to 1.9 times baseline or ≥0.3 mg/dl increase in serum creatinine or urine output < 0.5 ml/kg/hour for 6~12 h), stage 2 (2.0 to 2.9 times higher than baseline of serum creatinine or urine output < 0.5 ml/kg/hour for ≥12 h), and stage 3 (≥3 times baseline or increase of serum creatinine to ≥ 4.0 mg/dl or RRT or urine output < 0.3 ml/kg/hour for ≥24 h or anuria for ≥12 h).

### 2.3. Blood Sampling and Sex Hormone Measurement

Peripheral blood samples were collected 24 to 48 hours after the onset of MSAP and SAP. Serum levels of estradiol, progesterone, and testosterone were measured using commercially available immunoassays from the Clinical Laboratory Department of the Affiliated Hospital of Yangzhou University. C-reactive protein (CRP, mg/L), and white blood cells (∗10^9^/L) were analyzed according to certified standard analysis at the Clinical Laboratory Department of the Affiliated Hospital of Yangzhou University. Procalcitonin was measured using an accredited Elisa method based on monoclonal antiprocalcitonin antibodies and following routine methods at the Clinical Laboratory Department of the Affiliated Hospital of Yangzhou University.

### 2.4. Statistical Analysis

Normally distributed, continuous variables were summarized by reporting their means and standard deviations (SD) and were then compared with independent *t*-tests. Continuous variables which were not normally distributed were presented as medians and interquartile ranges (IQRs) and were then compared using Mann-Whitney *U* test. Differences in proportions were compared with Fisher's exact test or a *χ*^2^ test. Bivariate analyses were performed to identify factors potentially associated with AKI. Covariates found to be associated with AKI in the bivariate analysis with a *p* value of less than or equal to 0.20 were entered in stepwise backward multivariable logistic regression analyses with significance alpha levels less than or equal to 0.05 for retention. Results were shown as odds ratios (ORs) with 95% CI. The area under the receiver operating characteristic curve (AUCROC) was calculated in order to determine predictive abilities of sex hormone levels for the presence of AKI. A test was considered to be more accurate in the prediction of AKI if its AUCROC approached to 1.0, while it was little diagnostic value if its AUCROC approached to 0.5. Spearman correlation coefficients were reported for simple correlations. Statistical analyses were performed using SPSS (version 17.0; SPSS Inc., Chicago, IL, USA).

## 3. Result

### 3.1. Patient Characteristics

Data from a total of 122 patients with MSAP and SAP were collected. Thirty-three (27.05%) patients had AKI. The demographics, baseline characteristics, and hormone levels of patients classified by sex are shown in Table 1. There were 74 male patients (61%) and 48 female patients (39%) in total. The incidence of AKI between men and women was not significantly different (27.03 vs. 27.08%, *p* = 0.995). Regarding the sex hormones, also no differences were demonstrated in E2, progesterone, or testosterone levels.

The demographic characteristics, clinical parameters, and outcomes of patients classified by with or without AKI are shown in Table 2. No differences were observed in age, gender, and etiology between the AKI and non-AKI groups. However, comparing with patients without AKI, those with AKI had higher APACHE II (13.90 ± 3.64*vs*. 20.33 ± 2.73, *p* ≤ 0.01) and SOFA scores (7.11 ± 1.89 vs. 9.76 ± 3.30, *p* ≤ 0.01) as well as higher serum creatinine, C-creative protein, and PCT levels. In terms of outcomes, complications were higher for patients with AKI, particularly in septic shock and respiratory failure. Moreover, patients with AKI suffered longer ICU and hospital stay as well as a higher mortality rate (3.17% *vs*. 18.18%, *p* = 0.005).

### 3.2. Serum Sex Hormones between Patients with or without AKI

**Figure 1:**
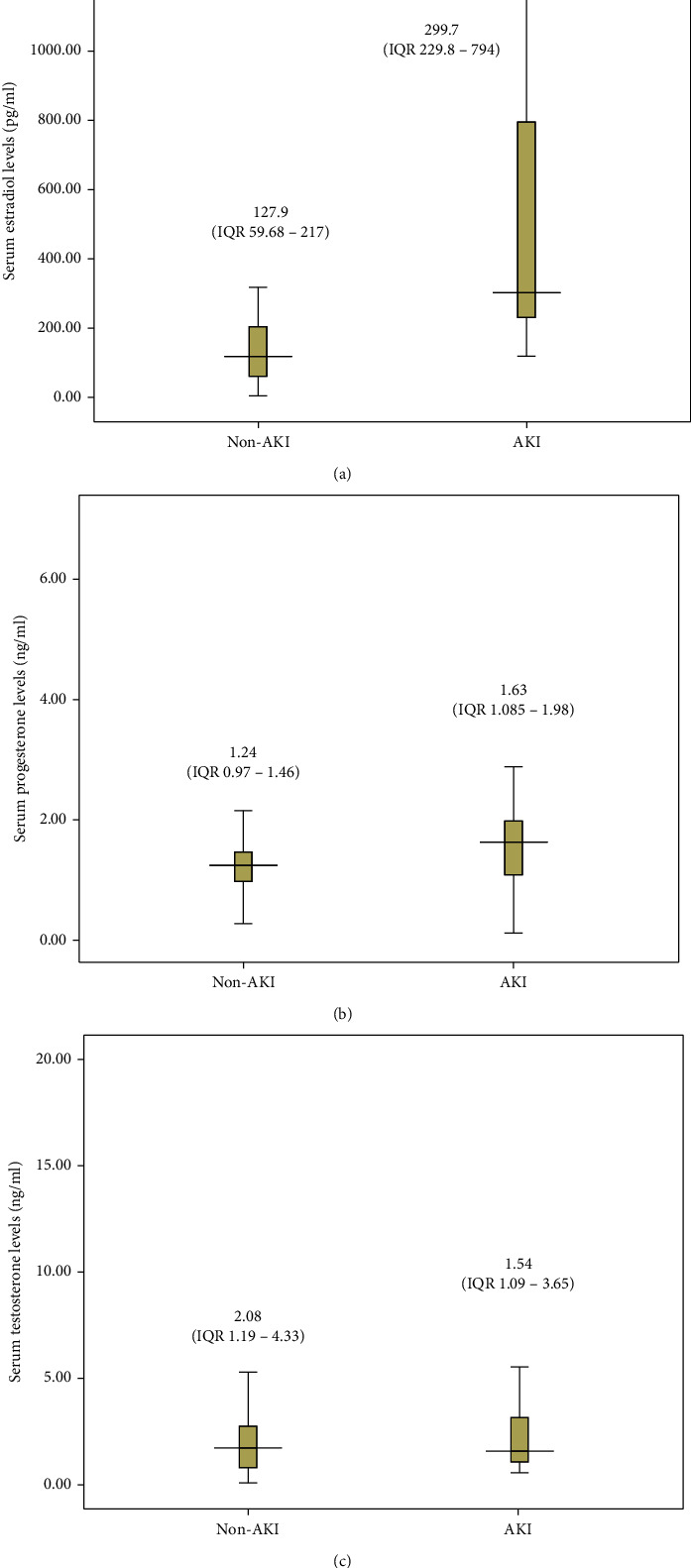
Comparison of serum sex hormone levels in patients with MSAP and SAP. (a) Estradiol and (b) progesterone and (c) testosterone levels in MSAP and SAP patients. The serum sex hormone level was determined during the first 48 hours following admission. Patients without AKI were identified by the left bars, and patients with AKI were identified by the right bars. Medians and interquartile ranges (IQR) were shown above each plot. Statistical significance was determined with the two-sided Mann-Whitney *U* test. Note: AKI: acute kidney injury; MSAP: moderately severe acute pancreatitis; SAP: severe acute pancreatitis.

Serum sex hormone levels during the first 48 hours following admission were comparable between patients with and without AKI. When compared with patients without AKI, those with AKI had higher estradiol levels (*p* ≤ 0.01) and slightly higher progesterone levels (*p* = 0.014) but similar testosterone levels (*p* = 0.668) (Figure 1).

### 3.3. Association between Serum Hormones and AKI in Patients with MSAP and SAP

Logistic regression analysis, which was associated with AKI, including age, gender, clinical complication, and serum sex hormone levels, is shown in Table 3. In the multivariable logistic regression model, after adjusting for covariates which were significantly associated with outcome, the serum estradiol level was found to be the only independent risk factor of AKI in MSAP and SAP patients with an odds ratio of 4.699 (95% CI 1.783-12.386, *p* = 0.002). In contrast, none of other related variables were independent risk factors for AKI. The APACHE II and SOFA scores were not included in our multivariate analysis as serum concentration of creatinine was already included in the scoring system. The model showed good calibration using Hosmer-Lemeshow goodness-of-fit test (*χ*^2^ = 6.027 and degrees of freedom =8, *p* = 0.644).

**Figure 2:**
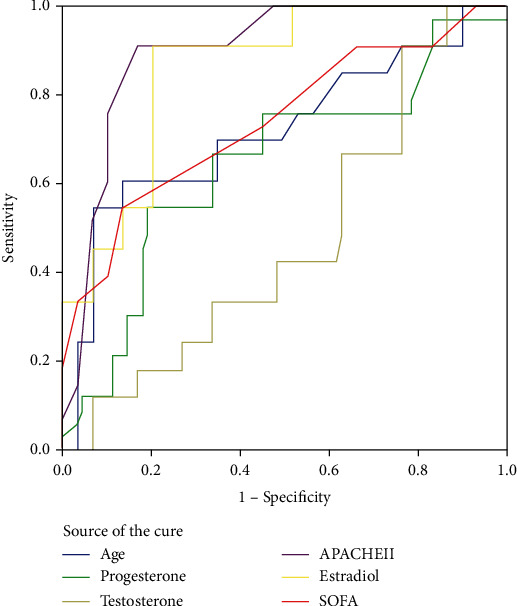
Receiver operating characteristic (ROC) curves of hormone levels, age, APACHE II, and SOFA scores for AKI in patients with MSAP and SAP. Note: APACHE II: Acute Physiology and Chronic Health Evaluation II; SOFA: sequential organ failure assessment; AKI: acute kidney injury; MSAP: moderately severe acute pancreatitis; SAP: severe acute pancreatitis.

As the observation of close correlation between serum sex hormones and AKI, we were then interested and further analyzed their predictive values for the presence of AKI in patients with MSAP and SAP. AUCROC analysis demonstrated that a serum estradiol cut-off point of 223.15 pg/mL pointed out optimal predictive value for the development of AKI with a sensitivity of 92.3%, a specificity of 79.3%, and an AUCROC of 0.859, which was better than other potential risk factors such as SOFA score (0.741) or age (0.719) (Table 4 and Figure 2).

### 3.4. Correlations between Serum Hormones and Selected Variables

Further analysis of the correlations between serum hormones and inflammatory parameters including WBC, PCT, and CRP were performed. Interestingly, this analysis established a linear relationship between serum estradiol level and CRP, suggesting the potential association between the elevated serum E2 level and increased inflammation (Table 5).

## 4. Discussion

### 4.1. Key Findings

In this study, we performed a retrospective study of patients with MSAP and SAP in order to investigate whether serum estradiol is associated with AKI or not in patients with MSAP and SAP. We found that, comparing with no-AKI patients, both serum estradiol and progesterone levels were significantly increased in MSAP and SAP patients with AKI. In multivariate analysis, increased serum estradiol level was an independent risk factor associated with the presence of AKI in patients with MSAP and SAP. However, there was no significant difference in testosterone level regarding the presence of AKI. In addition, escalated serum estradiol level was strongly correlated with increased CRP level.

### 4.2. Relationship of Findings to Other Relevant Studies

Both MSAP and SAP are clinically serious diseases along with multiple organ dysfunction diseases [1]. Kidney is the second most frequently damaged organ by MSAP and SAP [14]. Patients with both SAP and AKI have a higher mortality rate than those with SAP alone [15, 16]. In our study, we found that MSAP and SAP patients with AKI had longer ICU and hospital stays. In addition, the mortality rate was 18.18% in patients with AKI, which was much higher than those without AKI. Therefore, early prediction of AKI in MSAP and SAP patients may contribute physicians to perform appropriate therapy.

Although there are numerous studies focusing on screening novel biomarkers of AKI in SAP [7, 17, 18], most of them are investigated *in vitro* or *in vivo* models. Gender dimorphism has been extensively studied in sepsis and trauma [19–21]. The pathophysiology of AP is similar to those of sepsis, in that it rapidly leads to an overwhelming systemic inflammatory response and multiple organ failure [12, 22]. However, the impact of sex on mortality in AP patients remains controversial [23, 24]. Besides, the association between sex and acute kidney injury (AKI) in patients with acute pancreatitis is still unknown. Animal models have consistently shown a protective effect of female on the development of AKI after ischemia-reperfusion injury [6, 25, 26]. Contrary to these experimental observations, the direction of sexual dimorphism has been reported to be controversial in AKI in human [23, 27]. Some studies demonstrated that female was associated with improved survival in critically ill patients with septic acute kidney injury [28]. While Feng et al. showed that serum estradiol levels were predictive of 28-day mortality in pneumonia-related septic shock and were associated with concomitant AKI [8]. In fact, the studies mentioned above were conducted following different criteria to define AKI, which may contribute to conflicting results. Interestingly, a meta-analysis study found that the sex-stratified incidence of AKI varies according to the criteria used to define AKI [29].

Our study found no significant differences in incidences of MASP and SAP between men and women. However, serum estrogen levels were significantly increased in MSAP and SAP patients who had AKI. Lu et al. also demonstrated that the overall mortality rate for men and women in SAP patient were not significantly different. However, estradiol was significantly elevated in nonsurvivors [24]. These results indicated that sex hormones, rather than gender itself, were responsible for gender-specific findings [30].

The multivariate logistic regression model suggested that elevated serum estradiol levels were associated with an increased likelihood of developing AKI for MSAP and SAP patients. Furthermore, ROC curve analysis demonstrated that a serum E2 cut-off point value of 223.15 pg/mL had 92.3% sensitivity and 79.3% specificity for predicting AKI in MSAP and SAP patients, while the area under the AUC curve was 0.875. Our results are consistent with Feng et al. [8], who found that increased serum estradiol levels were associated with higher severity of concomitant AKI in patients with septic shock.

At present, the pathogenesis of SAP-induced renal injury remains elusive. It has been generally recognized that both proinflammatory and anti-inflammatory cytokines play a pivotal role in SAP-associated renal injury [30–33]. *In vivo* experiments have demonstrated that estrogen often has a modulation effect on the release of inflammatory cytokines [34–39]. However, clinical researches for adults demonstrated that no differences of proinflammatory cytokine such as TNF-*α*, IL-6, and IL-10 by gender were observed [19]. As the test of inflammatory cytokines in the MSAP and SAP patients enrolled in this retrospective study was not performed, we then analyzed the correlations between serum hormones and inflammatory parameters including C-reactive protein (CRP), an established inflammatory marker. Existing studies have shown that CRP is one of the key indicators for assessing the severity of SAP [40, 41]. A recent study demonstrated a marginal association between CRP and serum estradiol in middle-aged and older male populations [42]. Van Vught et al. [19] found high level of C-reactive protein in female sepsis patients on ICU admission. Consistent with these previous reports, our study demonstrated that escalated serum estradiol level was strongly correlated with increased CRP level but was not significantly correlated with WBC or PCT. Besides, progesterone and testosterone were also not significantly associated with CRP, PCT, or WBC.

### 4.3. Implications of Study Findings

Based on our findings, we suggest that serum estradiol during the first 48 hours following admission might be a key risk factor for AKI in patients with MSAP and SAP. For patients exhibiting high estradiol levels, clinicians should closely monitor renal function, avoid nephrotoxic agents, and consider early renal replacement therapy if renal function starts to deteriorate. In addition, our research found that escalated serum estradiol level was strongly correlated with increased CRP level, indicating a potential pathophysiology of SAP-related AKI. However, further pathophysiology studies are required.

### 4.4. Limitations

Along with all benefits of this study detailed above, it is worth to note the limitations. Firstly, the number of samples enrolled in this study is small, and all cases were selected from Affiliated Hospital of Yangzhou University; thus, the association between serum estradiol and AKI may not necessarily imply causality. Therefore, a larger number of samples and multicenter cooperation are required to further confirm these findings. Secondly, serum sex hormones were only measured during first 48 h, limiting the further evaluation of changing patterns of sex hormone levels during the course of MSAP and SAP in terms of predicting potential outcomes.

## 5. Conclusion

A high baseline serum estradiol level may be an independent risk factor of AKI in patients with MSAP and SAP. Serum estradiol level of 223.15 pg/mL is a good cut-off point for early identification of AKI in patients with MSAP and SAP.

## Figures and Tables

**Table 1:** Demographics and characteristics of patients classified by sex.

	Male (*n* = 74)	Female (*n* = 48)	*p* value
Age, yr, mean ± SD	53.08 ± 11.45	52.52 ± 10.01	0.782
APACHE II score	15.76 ± 4.36	15.46 ± 4.65	0.720
SOFA score	7.22 ± 1.53	7.94 ± 3.27	0.103
CRP, mg/L, median (IQR)	48.4 (12.99-80.39)	53.6 (13.59-114.58)	0.379
PCT, ng/ml, median (IQR)	0.86 (0.03-10.14)	2.5 (0.86-6.27)	0.083
WBC, ∗10^3^/*μ*L, mean ± SD	13.01 ± 5.00	14.39 ± 7.04	0.206
Estradiol (pg/mL)	169.80 (87.13-256.1)	217 (57.67-335)	0.519
Progesterone (ng/mL)	1.26 (0.87-1.62)	1.31 (1.15-1.91)	0.077
Testosterone (ng/mL)	1.74 (1.02-4.23)	2.09 (1.29-4.55)	0.261
Creatinine, mg/dL, mean ± SD	78.22 ± 10.78	86.96 ± 62.49	0.398
AKI, *n* (%)	20 (27.03)	13 (27.08)	0.995
Etiology of MSAP and SAP			
Biliary, *n* (%)	25 (33.78)	11 (22.92)	0.735
Hyperlipidemia, *n* (%)	31 (41.89)	27 (56.25)	0.121
Alcoholic, *n* (%)	14 (18.92)	2 (4.17)	0.018
Idiopathic, *n* (%)	4 (5.41)	8 (16.66)	<0.001

Note: MSAP: moderately severe acute pancreatitis; SAP: severe acute pancreatitis; AKI: acute kidney injury; SD: standard deviation; IQR: interquartile rate; CRP: C-reactive protein; PCT: procalcitonin; WBC: white blood cell count; APACHE II: Acute Physiology and Chronic Health Evaluation II; SOFA: sequential organ failure assessment. ∗ indicates statistical significance, *p* < 0.05.

**Table 2:** Demographic and clinical outcomes of patients classified by with or without AKI.

Variable	Overall	No AKI	AKI	*p* value
Patient numbers	*N* = 122	*N* = 89	*N* = 33	
Age, yr, mean ± SD	52.86 ± 10.87	51.71 ± 10.97	55.97 ± 10.09	0.054
Male, *n* (%)	74 (60.66)	54 (60.67)	20 (60.61)	0.995
Etiology				0.994
Biliary, *n* (%)	36 (29.51)	26 (29.21)	10 (30.3)	
Hyperlipidemia, *n* (%)	58 (47.54)	42 (47.20)	16 (48.48)	
Alcoholic, *n* (%)	16 (13.11)	12 (13.48)	4 (12.12)	
Idiopathic, *n* (%)	12 (9.84)	9 (10.11)	3 (9.10)	
Clinical parameters at 48 h				
Creatinine, mg/dL, mean ± SD	81.88 ± 15.59	53.60 ± 17.30	157.33 ± 12.64	<0.001^∗^
CRP, mg/L, median (IQR)	50.60 (13.26-98.65)	35.28 (12.34-58.10)	139.8 (65.54-154.2)	<0.001^∗^
PCT, ng/ml, median (IQR)	1.66 (0.12-6.79)	0.72 (0.03-2.35)	10.19 (4.83-26.5)	<0.001^∗^
WBC, ∗10^3^/*μ*L, mean ± SD	13.56 ± 5.90	12.04 ± 4.32	17.64 ± 7.54	<0.001^∗^
APACHE II score	15.64 ± 4.46	13.90 ± 3.64	20.33 ± 2.73	<0.001^∗^
SOFA score	7.83 ± 2.62	7.11 ± 1.89	9.76 ± 3.30	<0.001^∗^
Complications				
Septic shock, *n* (%)	13 (10.66)	6 (6.74)	7 (21.21)	0.021^∗^
Respiratory failure, *n* (%)	19 (15.57)	10 (11.24)	9 (27.27)	0.03^∗^
Clinical outcome				
28-day mortality, *n* (%)	9 (7.38)	3 (3.17)	6 (18.18)	0.005^∗^
Length of stay, dmean ± SD	15.85 ± 7.07	13.12 ± 5.02	23.21 ± 6.58	<0.001^∗^
Length of ICU stay, dmean ± SD	5.49 ± 5.05	3.61 ± 3.84	10.58 ± 4.40	<0.001^∗^

Note: MSAP: moderately severe acute pancreatitis; SAP: severe acute pancreatitis; AKI: acute kidney injury; SD: standard deviation; IQR: interquartile rate; CRP: C-reactive protein; PCT: procalcitonin; WBC: white blood cell count; APACHE II: Acute Physiology and Chronic Health Evaluation II; SOFA: sequential organ failure assessment. ∗ indicates statistical significance, *p* < 0.05.

**Table 3:** Binary logistic regression analysis of predictive factors for the complication of acute kidney injury in patients with MSAP and SAP.

Variables	Univariate analysis		Multivariate analysis	
	OR (95% CI)	*p* value	OR (95% CI)	*p* value
Age	1.063 (1.062-1.102)	0.001	1.052 (1.015-1.091)	0.006
Male gender	1.003 (0.443-2.273)	0.995		0.604
Sepsis shock	2.269 (0.821-6.268)	0.114		0.810
Respiratory failure	2.420 (0.999-5.861)	0.050		0.071
Estradiol (E2)	5.731 (2.246-14.622)	<0.001	4.699 (1.783-12.386)	0.002
Progesterone	1.823 (1.054-3.155)	0.032		0.158
Testosterone	0.955 (0.845-1.080)	0.464		0.524

Note: MSAP: moderately severe acute pancreatitis; SAP: severe acute pancreatitis; OR: odds ratio; CI: confidence interval.

**Table 4:** Diagnostic efficiency of age, APACHE II score, SOFA score, and serum hormone levels for of AKI in patient with MSAP and SAP.

	AUC	*p* value	Optimal cut-off points	Sensitivity, %	Specificity, %
Age	0.719	<0.001	66.5	54.5	93.3
APACHE II	0.898	<0.001	17	90.9	83.1
SOFA	0.741	<0.001	8.5	54.5	86.5
Estradiol	0.859	<0.001	223.15 pg/mL	92.3	79.3
Progesterone	0.645	0.014	1.625 ng/mL	54.5	80.9
Testosterone	0.475	0.668	1.63 ng/mL	48.5	37.1

Note: MSAP: moderately severe acute pancreatitis; SAP: severe acute pancreatitis; AKI: acute kidney injury; ROC: receiver operator characteristic; AUC: area under the receiver operating characteristic curve; APAPCHE II: acute physiology and chronic health evaluation score II; SOFA: sepsis-related organ failure assessment.

**Table 5:** Correlation coefficient analysis of serum hormones and the selected variables.

	WBC	CRP	PCT
Estradiol	0.123	0.796^∗^	0.328
Progesterone	0.245	0.359	0.101
Testosterone	-0.129	0.062	-0.109

Note: CRP: C-reactive protein; PCT: procalcitonin; WBC: white blood cell count. Values are spearman correlation coefficients. ^∗^*p* < 0.01.

## Data Availability

The data used to support the findings of this study are included within the article.
